# A Different Weight Loss Experience: A Qualitative Study Exploring the Behavioral, Physical, and Psychosocial Changes Associated with Yoga That Promote Weight Loss

**DOI:** 10.1155/2016/2914745

**Published:** 2016-08-10

**Authors:** A. Ross, A. Brooks, K. Touchton-Leonard, G. Wallen

**Affiliations:** Department of Nursing Research and Translational Science, National Institutes of Health Clinical Center, Bethesda, MD 20892, USA

## Abstract

Yoga interventions improve obesity-related outcomes including body mass index (BMI), body weight, body fat, and waist circumference, yet it is unclear whether these improvements are due to increased physical activity, increased lean muscle mass, and/or changes in eating behaviors. The purpose of this study is to expand our understanding of the experience of losing weight through yoga.* Methods.* Semistructured interviews were qualitatively analyzed using a descriptive phenomenological approach.* Results.* Two distinct groups who had lost weight through yoga responded: those who were overweight and had repeatedly struggled in their attempts to lose weight (55%, *n* = 11) and those who were of normal weight and had lost weight unintentionally (45%, *n* = 9). Five themes emerged that differed slightly by group: shift toward healthy eating, impact of the yoga community/yoga culture, physical changes, psychological changes, and the belief that the yoga weight loss experience was different than past weight loss experiences.* Conclusions.* These findings imply that yoga could offer diverse behavioral, physical, and psychosocial effects that may make it a useful tool for weight loss. Role modeling and social support provided by the yoga community may contribute to weight loss, particularly for individuals struggling to lose weight.

## 1. Introduction

Obesity, defined by the Centers for Disease Control and Prevention (CDC) as a body mass index (BMI) of 30 or greater [1], is epidemic in the USA and plays a pivotal role in many chronic health conditions [2, 3]. Greater than 30% of the US population (an estimated 72.5 million) is obese, at an annual cost of $147 billion dollars in medical costs [4]. A number of elements contribute to the obesity epidemic, but the Surgeon General has cited three main factors that play an important role: decreased physical activity; increased consumption of high caloric, high fat and nutrient-poor foods; and stress [5]. Strong evidence shows that a dose-response relationship exists between stress and abdominal adiposity and obesity [6]. Stress also affects food-seeking behaviors including increased consumption of foods high in fat and sugar [7, 8].

No single solution for reducing obesity exists. Over 300,000 bariatric surgeries were performed worldwide to treat obesity in 2011 and while potentially effective in reducing body weight and prolonging survival, these surgeries pose significant risk for complications [9]. Traditional weight loss programs focusing on diet and exercise to produce an energy deficit frequently result in weight loss, but long-term weight maintenance remains elusive [10]. Few of these treatments address the complex psychological and behavioral issues that initially led to weight gain.

Yoga, an ancient discipline involving physical poses, breath work, and mindfulness techniques, is the most commonly used nondietary or supplement complementary and alternative therapy for weight loss [11]. In clinical trials, yoga has improved a number of obesity-related outcomes including BMI, body weight, body fat, and waist circumference [12]. Individuals who practice yoga report that yoga helps to improve diet and body weight [13], and studies involving long-term yoga practitioners have found an inverse relationship between frequency of yoga practice and levels of obesity [14]. In the population-based, longitudinal vitamin and lifestyle (VITAL) study of 15,550 adults, individuals who practiced yoga for at least four years were two to four times less likely to gain weight as they aged than individuals who did not practice yoga [15]. In a review of 55 research studies examining yoga for weight-related outcomes, Rioux and Ritenbaugh (2013) found yoga interventions to be effective for achieving weight loss and improving body composition [12]; the most effective programs were residential and longer in duration, required more frequent and home practice, included a yogic diet, and incorporated a variety of yoga practices as opposed to exclusively focusing on a single practice such as the physical poses or breath work.

The mechanism underlying yoga's effectiveness at improving weight-related outcomes remains unclear, although a number of pathways have been proposed including increased energy expenditure, reduced pain, enhanced mindfulness and body awareness, and reduced stress [16]. Yoga appears to downregulate the Hypothalamic-Pituitary-Adrenal (HPA) axis and the Sympathetic-Adrenal-Medullary (SAM) response to stress [17]. Additionally, yoga interventions have been shown to reduce binge eating and preoccupation with food [18, 19]. Adipose tissue acts as an endocrine organ, secreting adipokines that impact energy intake, fat storage, and metabolism such as adiponectin, which is anti-inflammatory, enhances insulin sensitivity, and is inversely associated with obesity, as well as leptin, which is highly correlated with obesity, insulin resistance, and type 2 diabetes [20]. In a cross-sectional comparison study involving 25 novice female yoga practitioners and 25 expert practitioners matched for age, fitness level, and abdominal adiposity, levels of leptin were 36% higher, and levels of adiponectin were 28% lower, in the novice group than in the expert group [21], suggesting that long-term yoga practice may affect metabolism. While yoga interventions may promote weight loss and improve body composition, nearly all of the research has been quantitative in nature, primarily examining whether a given yoga intervention results in weight loss. Of these, many utilize small sample sizes and weak methodology [16]. In those studies that have resulted in weight loss, it is unclear whether weight loss is due to increased physical activity, increased muscle mass, psychosocial factors, and/or changes in eating behaviors. The primary aim of this study is to expand our understanding of the experience of losing weight through the practice of yoga. The primary research question is as follows: “what is the experience of individuals who have lost weight and believe that yoga practice contributed to this weight loss?”

## 2. Methods

### 2.1. Participants, Recruitment, Ethics, and Consent

After receiving approval from the National Institutes of Health Clinical Center's (NIHCC) Office of Human Subjects Research Protection (OHSRP), investigators worked with the Iyengar Yoga National Association of the United States (IYNAUS) to locate yoga studio owners who might know individuals who had lost weight through the practice of yoga. Iyengar yoga was chosen because it has a highly structured national organization. In addition, unlike some schools of yoga that focus exclusively on one aspect of yoga such as the physical postures or breath work, Iyengar yoga is a classical form of yoga with strict standardization of teaching that emphasizes a number of aspects of yoga including the physical poses, breath work, and philosophy. Studio owners were emailed a letter providing information about the study and seeking help identifying individuals who had lost weight and believed that their yoga practice had contributed to their weight loss. The studio owners posted information about the study in their studios and/or in their newsletters, along with contact information for the investigators. Individuals who contacted the researchers and expressed interest in participating in the study underwent a telephone screen to assess for the eligibility criteria. Individuals were included if they were (1) 18 years or older and (2) a yoga practitioner who identified himself or herself as having lost weight through the practice of yoga. As per the approved protocol, verbal consent was obtained prior to each interview. Based upon a review of the literature regarding phenomenological approaches, a sample size of 20 was sought [22–24].

### 2.2. Data Collection

If the individual met the inclusion criteria, two semistructured telephone interviews were scheduled. In the initial interview participants were encouraged to explain their experiences with losing weight through the practice of yoga in as much detail as possible. All interviews were audiotaped and then downloaded onto a secure computer and transcribed verbatim. Within two weeks of the initial interview, the primary investigator read and reread the interview, extracting significant statements that captured the essence of the interview. Then, a short follow-up telephone call was made to confirm the findings of the interview and to offer participants an opportunity to add any additional experiences they did not think of during the first interview or to retract comments that they felt did not represent their views. These changes were incorporated into the subject's data. Having participants confirm a synopsis of the transcript and allowing subjects to add or retract comments in the follow-up interview allowed the researchers to better describe the experience and added rigor to the study [25].

### 2.3. Data Analysis

Qualitative data were analyzed using a descriptive phenomenological approach, as outlined by Colaizzi [26, 27]. Audio files were transcribed verbatim, and then a research team member performed an internal reliability check by listening to all audio files to confirm the interviews were correctly transcribed. Two investigators independently read and reread the transcripts to obtain a feel for the participants' experiences. They then extracted statements that described the phenomenon and illuminated the experience. They formulated meanings from these statements and assigned those meanings codes, words, or phrases that describe and summarize the meaning of the subject's experience. These codes were placed into themes, represented by categories of experiences that were universal to the participants that then were unified into a comprehensive description of the phenomenon. To assist in bracketing, not allowing prior experiences and beliefs to interfere with data analysis, one coder was included who had no prior connections to yoga. During the iterative coding process, resolution of discordant meanings and minor reorganization of the thematic structure occurred through consensus.

To ensure trustworthiness of the data, three criteria for assessing rigor were considered: credibility, auditability, and fittingness of data [28]. Credibility was established by having two team members analyze the data independently and then having a third study team member with expertise in qualitative methodology validate the themes and coding. For auditability, examples of data are presented alongside each theme and subtheme to illustrate how data led to each theme being identified [25]. To address fittingness, 80% of participants were interviewed at two time points, providing a wealth of data from which to draw inferences and describe the experience [25]. NVivo (QSR International Pty Ltd. Version 10.0, 2012) was used for qualitative data analysis and management and to establish two measures of interrater reliability, a kappa coefficient and percent agreement. A kappa coefficient of  0.85 and percent agreement of 99.2% were calculated for this study. Descriptive statistics were calculated using IBM SPSS (Statistics for Windows, Version 22.0).

## 3. Results

Twenty-one subjects were screened, and 20 subjects participated in the semistructured interviews and are included in these analyses (Table 1). Sixteen participants (80%) were available to participate in a second interview. The initial interviews ranged between 9.3 and 45.1 minutes (m = 26.6 ± 9.5 minutes), and follow-up interviews ranged from 4.3 to 25.6 minutes (m = 12.6 ± 5.6 minutes). Mean age of participants was 56.6 (±9.4) years. Approximately three-quarters of the subjects were female (75%) and married (*n* = 14, 70%). Nearly all were white and non-Hispanic (95%). They were highly educated, with nearly three-quarters having a M.S. (30%) or Ph.D. (40%) degree. The majority (85%) of participants practiced Iyengar yoga, either exclusively (75%) or in combination with another style of yoga (10%). Subjects had practiced yoga between one and 45 years (mean = 15.3 ± 13.6) years. They reported an average weight loss of just over 26 pounds (range = 4–70). Prior to losing weight, just over half of subjects were overweight or obese (55%; *n* = 11), with a mean BMI of 27.4 ± 5.7 (range = 17.1–41.8). Current BMIs ranged from 16.4 to 31.5, with a mean of 23.2 (±3.2), placing the majority (75%) of subjects into the normal range for BMI.

Two distinct groups of individuals responded to the recruitment emails and participated in the study: those who were overweight when they started yoga (55%, *n* = 11), many of whom had repeatedly tried and failed to lose weight, and those who were of normal weight (plus one below normal), nearly all of whom had lost weight unintentionally (45%, *n* = 9). Several key themes emerged from the interviews including a shift toward healthy eating, the impact of the yoga community and yoga culture, physical changes associated with yoga, psychological changes associated with yoga, and the belief that the yoga weight loss experience was different than past weight loss experiences. While some of the themes and subthemes were universal, some differed slightly based upon the group to which individuals belonged (Table 2). The themes and subthemes, as well as the group differences, are discussed in the sections below.

### 3.1. Theme One: Shift toward Healthy Eating

Ninety percent of subjects (*n* = 18) reported a shift toward healthy eating that could be categorized into three distinct subthemes: an increase in mindful eating, changes in food choices, and decreased emotional and/or stress eating. Fifteen of the 20 subjects discussed the belief that yoga practice led to an increased sense of mindfulness or awareness regarding what they ate and the circumstances surrounding their food consumption.

*I wouldn't say I was really dieting. I was just being very conscious of changing the way I related to food. And part of changing the way that I related to food involved just paying attention to what I eat and when I eat, and you know, I think I began to think of eating more as dining and less as fueling up.* (60-year-old male, overweight before yoga)

Many subjects who previously struggled with their weight discussed how they no longer found themselves mindlessly eating or making unconscious food selections and purchases.

*…You could eat a bag of potato chips and not be aware of it… People can sort of zone out and they don't even know how much they are eating. Yoga definitely helps make you more mindful.* (44-year-old, female, overweight before yoga)

*I don't do drive-thru's [sic]. I can walk through the bakery in any grocery store. I have no triggers after yoga.* (60-year-old female, overweight before yoga)

In addition to being more aware of what and how they were eating, there was a practical aspect of yoga reported by subjects in both groups in that they became aware of the negative effect that eating too much food had on their yoga practice.

*Overeating also affects my practice. It's very hard to overeat the night before and get up the next morning and do a yoga class, because you have the feeling of you're still digesting.* (61-year-old female, overweight before yoga)

In the past, some participants reported exercising in order to be able to eat a certain number of calories, while now they ate to improve their general health and yoga practice.

*So now instead of eating food as this reward post-exercise, like exercising to eat, I concentrate more on making healthy food choices to, I guess, feed my body in the right way so that it will help my yoga practice…If I was just exercising…and you can see on the machine the number of calories you burn…I was like, ‘oh great, 600 calories.' I could just eat whatever I want. There don't seem to be any other consequences [of overeating]. It's not going to make me bad in spin class next time I go.* (35-year-old female, overweight before yoga)

They became aware that their practice was impacted not only by the amount of food they ate, but also by the type of food eaten. Subjects reported becoming aware of the effect of certain foods on their bodies during their yoga practice, including sugar, dairy, meat, and alcohol.

*I could feel how eating meat made me feel sluggish and hard to work in poses as opposed to when I wouldn't.”*(64-year-old female, normal weight before yoga)

*I found that if I had dairy the yoga was harder.* (44-year-old female, normal weight before yoga)

*Now I noticed that like, if I have a class on Sunday morning and even if I have a few glasses of wine on Saturday night, I might have just a slight headache on Sunday morning, so I just don't bother. You know, it's not a big deal to me one way or the other, but it's worth having a good class to not do anything that's not very important to me that's gonna kind of impact class.* (62-year-old female, normal weight before yoga)

In addition to avoiding certain foods and alcohol because of their impact on their yoga practice, a number of practitioners reported developing actual aversions to certain foods and alcohol.

*Well, I have noticed a big change in my food. I used to like to eat a lot of sweets and I've just lost the taste for that. And I used to get up in the morning and have a couple of strong lattes to get me going, and I've lost the need for that since I've started pranayama* [yogic breathing]* about a year ago. So yeah, my diet has changed. And alcohol, I've kind of had a loss of interest in having a cocktail or a glass of wine on a regular basis. In fact, it's almost like I have an aversion to alcohol at this point. I just don't like the feeling of it.* (61-year-old female, underweight before yoga)

Food cravings, in general, decreased in a number of practitioners.

*Well, with all of these things* [alcohol, sugar and caffeine],* I no longer crave some. I think because I became more aware of the downside of them.* (61-year-old female, underweight before yoga)

While one underweight subject reported that yoga practice led to an increase in appetite, a quarter of the participants specifically mentioned that yoga decreased their appetite.

*I find my appetite goes down. I haven't been eating as much, getting back into yoga.* (39-year-old female, overweight before yoga)

The belief that yoga reduces swings in blood sugar and therefore reduces sugar cravings and overeating was a common one.

*It's interesting. I can go into a yoga class feeling kind of hungry. Maybe I go before breakfast or around breakfast time, and I come out of class, I am not really hungry, and I think it's just because for some reason, blood sugar evens out in yoga. I have definitely noticed that it moderates hunger, and then it sort of gives me time, because my blood sugar isn't just plummeting…You don't have these blood sugar swings that would drive me just to grab the first thing I see…If your blood sugar is dropping, you're not in a good mood, and you're not really in control as much.* (62-year-old male, overweight before yoga)

In addition to variations in blood sugar impacting their food choices, many of the initially overweight subjects discussed a direct relationship between their moods and stress levels with their food consumption.

*Not only how many calories you put in but how much food you actually eat is really determined by your emotional state. So I …had a big habit of eating whenever I was stressed…I would eat to calm myself, and I would eat when I was bored. I'd eat when I was lonely. I'd eat when I was too excited. I ate for social reasons; I mean every reason in the book.* (61-year-old female, overweight before yoga)

Participants discussed how this pattern of eating due to stress or negative emotions lessened after starting to practice yoga.

*My emotion eating. There was less that I needed. There was less emotion that I needed to feed.* (44-year-old female, normal weight before yoga)

While the yoga may not have eliminated unhealthy eating patterns, it helped to improve them.

*That it did help me with recognizing, like, ‘oh, you are an anxious eater.' And I still did that when I started practicing yoga. I did have an eating disorder and I still struggle with it a little, but not as badly because yoga helped me calm the anxiety so that food was not so numbing. I don't have to numb through food. I could use the yoga to calm.* (44-year-old female, overweight before yoga)

Not only did yoga reduce unconscious and emotional eating, but participants also believed that the stress reduction associated with yoga put them in a better frame of mind to make healthier food choices.

*When I'm doing yoga, I'm not as anxious. Everything is a little slowed down. I can go in there [*the cafeteria*] and I can organize things better and think better, and I'll say, ‘Ok. Well, they have fish and they've got rice, macaroni and cheese, and all this stuff, but they also have green beans, and I can eat those today. So I'm going to eat the fish, and I'm going to get green beans and drink some water. And I'll eat it, and I don't want anything more. And I can just kind of go back to work. While, if I'm not doing yoga and I'm just really stressed and very focused, and the whole day is rushing by, I might eat and then I'm still hungry. And I might eat some chocolate or something else, and I'll need that extra bit of sugar to sort of propel me through the rest of the day. I find that I don't eat that when I'm doing yoga.* (39-year-old female, overweight before yoga)

### 3.2. Theme Two: Impact of Yoga Community/Yoga Culture

A second theme that emerged from the interviews of sixty percent of participants (*n* = 12), particularly those who were initially overweight, was that the yoga community and yoga culture played a role in their weight loss. Two subthemes emerged, including that their yoga teachers and more advanced yoga practitioners served as role models for healthy behaviors and that there was a different attitude and sense of support among the yoga community than that which is found in the culture of many gyms or health clubs. Participants believed that both of these aspects of social support contributed to their weight loss.

Participants discussed being inspired to live a healthier lifestyle by their peers and, even more so, by their yoga teachers, who served as role models for healthy living. For some, the inspiration they received from their teachers was profound, and it influenced their food choices.

*I was shocked, totally shocked, to see my teacher move…when I saw her move in the sun salutations and she did this headstand with lotus and these backbends that were just beautiful. I just couldn't believe my eyes. I don't think it was possible to, you know, have that kind of capability…I began to emulate my teacher, and I think that she inspired me to think twice about what I was eating.* (64-year-old female overweight before yoga)

A number of participants specifically discussed how just being in the presence of individuals in the yoga community and witnessing their behaviors influenced their own health behaviors and food choices.

*Yoga, the practice itself and the people who teach it and do it, have a healthier world view and way of looking at life, and so you become the people you're around, and so it just helps.* (44-year-old female, normal weight before yoga)

Not only did the individuals within the yoga community serve as role models for healthy behaviors, but also they also were role models for taking care of oneself, which translated into making healthier food choices. The yoga community conveyed an attitude of kindness, acceptance, and support, and participants discussed how this attitude transferred to how they treated themselves.

*Being in that culture, that society, of being around people who are like, ‘be good to yourself', ‘be kind to yourself', it changes your thinking as well so that… I'm not, ‘oh I'm such a horrible person and I'm going to go eat some potato chips.* (44-year-old female, normal weight before yoga)

Participants perceived this attitude as being distinctly different than the attitude and mindset that participants had experienced at the gym.

*…takes the competitive thinking out of the game and when you go to yoga, at least in the studio where I practice, it's a very safe place…You walk up the stairs and you close the door and go into your studio and you're not competing with everybody else. There's a gentleness in there and you can really just focus on yourself and practice leaving what else is going on in your life, leaving it somewhere else and just focusing on yourself, and that's really rewarding.* (59-year-old female, overweight before yoga)

Several participants commented that the safety and comfort they felt during yoga may be related to the fact that, unlike the gym, not everyone in a yoga class was thin and fit, allowing them to feel comfortable participating. A number expressed the belief that the type of yoga classes one attends would make a difference as to whether it would support one's efforts at weight loss.

*You know, I don't see a lot of hard bodies. There's not what I would call a hard body in the yoga classes. They are not gym bunnies. They're normal people with a soft outer layer of fat. But they're healthy, you know, and they're all different sizes. I felt comfortable even when I was close to 200 pounds…. There were no model-looking like people, there were no midriffs showing, just a bunch of normal… now again, because I picked that kind of studio. I have seen studios that are a whole lot like the gym workout; it's like an aerobics class instead of a yoga class. Where the gym hosts you know, a yoga class, amongst all their other aerobic lineups, that's a different environment. For me, I don't think I would have had the experience or the success that I've had, had I just joined the gym and hit the weekly class at the gym.* (44-year-old female, normal weight before yoga)

### 3.3. Theme Three: Physical Changes

All 20 participants spoke of physical changes associated with yoga practice that they believed contributed to their weight loss. Several subthemes emerged including increased muscle tone, changes in metabolism; specific effects of yoga poses and yoga practice on weight; and other general physical changes that contributed to weight loss.

When asked how yoga helped them to lose weight, the most common answer from all practitioners was through the building and toning of muscle.

*I think yoga helps to develop and tone muscle. My muscle mass is definitely higher now than it was before, so I think that obviously contributes to weight loss.* (61-year-old female, overweight before yoga)

A number believed that the increase in muscle mass caused an increase in metabolism.

*On a basic level, it builds muscle, which burns more calories. So I think that, in itself, the way it tones muscles, just in my daily life, after I'm toning my muscles, then I can go out and I'm moving and burning more calories.* (44-year-old female, overweight before yoga)

In particular, practitioners of Ashtanga and Bikram styles as well as more vigorous forms of Iyengar yoga spoke of an aerobic effect that burned calories.

Y*ou are using a lot of energy as you're moving through the poses, so it is a form of exercise, and sometimes aerobic exercise, because of certain kinds of sequencing of poses.* (61-year-old female, normal weight before yoga)

Among nearly all of practitioners whose BMI fell within the normal category (88%), as well as several who were initially overweight (27%), the weight loss was unintentional. Three of the normal weight practitioners were so alarmed by their weight loss that they consulted their physicians. A number of practitioners believed that performing specific poses contributed to their weight loss. Among those poses that were most commonly cited as contributing to weight loss were twisting poses as well as inverted poses such as head stand and shoulder stand. Almost half of participants (*n* = 9) mentioned losing weight from specific areas of their body, and eight of these nine subjects reported losing weight from their waist or midsection.

*The weight loss was specific to my abdomen.* (59-year-old male, normal weight before yoga)

In addition to losing weight when they began to practice yoga, a number of subjects reported that their weight increased when their yoga practice was interrupted for a period of time, highlighting an inverse relationship between yoga practice and weight.

*I stopped doing yoga for a few years and slowly gained more and more weight. And then last year I decided to start doing yoga again. I'd already lost about 20 pounds through diet alone, but I started to do yoga again and probably lost another five to ten pounds.* (39-year-old female, overweight before yoga)

Subjects reported a number of general physical improvements that coincided with yoga practice and contributed to their weight loss. Among these physical changes were improved sleep and decreased pain. In addition, nearly every subject (90%) talked about feeling better physically, with most mentioning feeling more energized, which in turn allowed them to be more active.

*I feel more energetic. I can walk better. I can reach things better, so it helped with flexibility. I was better able to move my body and in general get through the day easier.* (44-year-old female, overweight before yoga)

### 3.4. Theme Four: Psychological Changes

Nearly all of the practitioners (95%) discussed psychological and mental changes associated with yoga practice. Particularly among the practitioners who were initially overweight, they believed these changes played a role in their weight loss. Within this theme, a number of subthemes emerged: a shift in mindset away from weight loss and towards health; spirituality; increased mindfulness and focus; improved mood and emotional stability; reduced stress; and increased self-esteem and self-acceptance. A number of subjects specifically reported a shift in mindset after starting yoga that contributed to their weight loss.

*It was just as my mindset shifted, as I made health a priority and not weight a priority, my diet changed.* (44-year-old female, normal weight before yoga)

Several participants specifically emphasized that yoga was more than a physical practice, with nearly 50% of subjects mentioning spirituality and/or spiritual effects.

*It's not just a physical practice. If you want to practice yoga, you are going to do more than the poses. That's the real practice. I mean, that's what it encompasses, the spiritual practice.* (63-year-old female, normal weight before yoga)

Among the psychological changes they noted were increased mindfulness, clarity, focus, and discipline.

*I was thinking the main focus for me is that it's made me more body aware and made me more mind aware, so I have been able to make better choices.* (62-year-old male, overweight before yoga)

This newfound awareness extended to body awareness. This was a new experience for the initially overweight subjects, many of whom reported past problems with proprioception, the sense of where one's body and body parts are oriented in space.

*The practice has really helped me become very aware of my body.* (61-year-old female, overweight before yoga)

The subjects also discussed improved mood, greater emotional stability, and less reactivity.

*I walked out and I felt so balanced and mellow and just good from the very first session. So I would say I felt the emotional, the psychological side of the yoga experience immediately.* (60-year-old male, overweight before yoga)

Yoga also provided an outlet for dealing with stressful situations and people that contributed to weight loss, particularly for the subjects who were overweight.

*I'm much more able to deal with my mother…It allows you to protect yourself. That's part of the eating thing too. You know with yoga you can protect yourself from forces, so instead of having a conversation with my mother that frustrates me and makes me angry which sends me to the mac and cheese, I don't do that anymore.* (59-year-old female, overweight before yoga)

Other psychological changes that bolstered weight loss, particularly for the initially overweight subjects, included increased self-esteem, self-confidence, and self-acceptance.

*It's increased my confidence, but more than that, it's made me satisfied with what I am. So I'm not looking to be something other than who I am, and that was what kind of freed me up to say, ‘OK. I can do this. I can lose this weight.* (59-year-old female, overweight before yoga)

This improved sense of self helped the participants who were initially overweight begin to take better care of themselves.

*It made me satisfied with who I am, so I stopped doing bad things to myself, like eating really bad stuff…You stop punishing yourself. I think poor eating is a little bit of punishing yourself. You know, if you don't like who you are, then you don't really care if you're fat.* (59-year-old female, overweight before yoga)

Particularly for the subjects who were initially overweight, many of whom reported past problems with proprioception and body dysmorphia, the newfound body awareness and acceptance were profound.

*I rarely have experiences of body dysmorphia, and I know this is a huge thing with many women…I haven't had this experience, and I think it's because I have this level of comfort in my body that I have never had before. I have this centeredness in my body that doesn't feel like a passing thing.* (60-year-old female, overweight before yoga)

It should be noted that two themes, “a shift toward healthy eating” and “psychological changes,” would frequently overlap and were often double coded. Subjects would often begin discussing psychological changes associated with yoga and then make a conscious or unconscious transition into discussing an increase in mindful eating or a decrease in cravings or stress-related eating.

### 3.5. Theme Five: A Different Weight Loss Experience

Nine out of the eleven subjects who were overweight or obese when they started yoga practice discussed trying unsuccessfully to lose weight in the past. For these subjects, this weight loss experience was distinctly different than past weight loss experiences. For many, the primary difference was that this weight loss was often unintentional and, unlike in the past, not difficult.

*It was the easiest experience for weight loss that I have ever had. This time it was just, I don't know, it was totally different for me.* (60-year-old female, overweight before yoga)

A number of the subjects reported that they struggled with adhering to formal weight loss plans in the past, and they believed yoga gave them the strength and put them in the proper mind set to adhere to their diet or formal weight loss program.

*I was on a medically supported program of weight loss…but it was yoga that put me in the mindset that I could possibly even try to do this…No, I wouldn't have had the mental strength to do that in the past…* (59-year-old female, overweight before yoga)

Nearly all of the eleven subjects who were initially overweight reported a past pattern of losing weight through diet and/or exercise, only to regain it.

*I was always a yo-yo dieter before. I was 40 pounds up, 40 pounds down. It was always the same 40 pounds.* (60-year-old female, overweight before yoga)

Unlike in the past,all of the subjects reported maintaining the weight lost through yoga practice, many for decades.

*I have kept it off for quite some time. You know, I feel pretty stable now and that's the yoga.* (61-year-old female, overweight before yoga)

A quarter of subjects emphasized the difference between yoga and standard exercise, as it pertains to weight loss.

*I have always hated going to the gym…all through my attempts to lose weight, I would go to the gym and go home and eat whatever I felt like. So it was always very self-defeating. It was a vicious cycle of, ‘Oh, I'll go exercise, and I'll go eat.' So, that's gone. I get this very clear physical power when I finish a class and it is combined with a deep relaxation…I don't think you get that from the fitness industry. I mean, you get a routine.* (60-year-old female, overweight before yoga)

## 4. Discussion

The demographic characteristics of this sample of yoga practitioners are similar to those found in national surveys of yoga practitioners in the USA and abroad [13, 29], with the majority being white, educated females. The themes that arose in this study were similar to those found by McIver et al. (2009), who qualitatively analyzed journals of 20 obese women attending a 12-week yoga treatment program for binge eating and found that the yoga program led to a healthy reconnection to food, physical empowerment, and increased awareness [19]. The concept of mindfulness was pervasive in this study, as many subjects reported an increase in mindful awareness regarding the food they ate, the emotions they felt, and the connection between the two. Mindfulness interventions may be effective additions to traditional weight loss programs, as a review of the literature on mindfulness and weight loss found that mindfulness interventions resulted in significant weight loss in 13 of the 19 studies reviewed [30]. Authors of this review suggested that mindfulness may play a role in weight loss by increasing one's self-regulatory capacity, increasing awareness of negative emotions and social cues that trigger overeating, and facilitating tolerance for discomfort [30]. The themes that emerged from the participants in this study, particularly those who were initially overweight, supported these conclusions.

Stress was a concept that was discussed repeatedly in this study, as many brought up the relationship between stress and binge eating and the role of yoga in relieving stress and stress-related eating. Evidence supports that stress and the resultant release of cortisol are associated with increased consumption of high fat, high-sugar foods [7, 8]. Yoga appears to decrease stress by downregulating the HPA axis response to stress. Perhaps this is the pathway through which yoga reduces stress-related eating. Because nearly half of the subjects reported losing weight specifically in their abdomen and there is a strong relationship between stress, cortisol release, and abdominal adiposity [6], the abdominal weight loss may also be an indication of decreased HPA axis activation.

A novel theme that emerged in this study was the influence of the yoga community and yoga culture on weight loss. Epidemiological data from the Framingham Heart Study showed that obesity appears to spread through social ties, with one's odds of becoming obese increasing if one's spouse, siblings, and most importantly one's friends became obese [31]. The authors suggested that one possible explanation for the contagious nature of obesity is the underlying norms regarding the acceptability of being overweight and/or consuming unhealthy foods. The authors concluded that social networks potentially could be used to spread positive health behaviors, a phenomenon that appears to have occurred in this study. Not only do social networks influence health behaviors, but social support also plays a critical role in weight maintenance [32]. Participants discussed the supportive, friendly environment of the yoga studios, and many of the initially overweight subjects believed that the social support and acceptance that they received were critical to their weight loss success. Likewise, a number emphasized that practicing yoga in studios or gyms that encouraged an atmosphere of competition or that attracted primarily thin, fit yoga practitioners would have been counterproductive to their weight loss attempts. There appear to be differences in yoga practitioners who take classes in gyms versus yoga studios, as Dittmann and Freedman (2009) found that yoga practitioners who take classes in gyms were primarily motivated to practice for physical and appearance reasons, while practitioners who took classes in yoga studios were primarily motivated for psychospiritual reasons [33].

While many commercial weight loss programs focus on an equation of calories in and calories out, weight gain likely is more complicated and includes physiological, social, environmental, and psychological factors such as stress, anxiety, and depression [34]. The participants viewed yoga as having a broad range of effects that contribute to weight loss. These effects would overlap, as a number of the themes frequently emerged simultaneously in the transcripts. For example, psychological changes such as increased mindfulness, improved mood, or reduced stress would often precede or be double coded with the theme of healthy eating, indicating that they were intertwined, at least in the thoughts and words of the participants.

There are a number of limitations of this study. As with any study using purposive sampling, the risk of selection bias could lead to a nonrepresentative sample. While the researchers conducting data analysis attempted to use bracketing to minimize their own bias, personal bias could affect coding. Another weakness of the study is the small sample size. While twenty subjects are not small for a qualitative study, only 10 participants from each of the groups were interviewed. Greater insight might be gained by interviewing more individuals in each of the two groups. However, this study has a number of strengths including the novelty of this underexplored topic, the rigor of the qualitative analysis and validation process, and the individual variation within the subjects that allowed unique experiences and subgroups to emerge.

Further research is needed to examine the physiological, psychological, and behavioral pathways whereby yoga leads to weight loss, including uncovering the mechanisms that explain how yoga decreases stress eating and reduces waist circumference. Research is needed to determine whether yoga can stand alone as a method of weight loss or should be used in combination with other weight loss interventions such as diet and exercise. The majority of the previously overweight subjects used a combination of diet and yoga to lose weight, while the weight loss of the subjects with normal BMI levels tended to be more unintentional and was not paired with conscious changes in diet. Further research is needed to ascertain the differential effects of yoga on both normal weight and obese populations, as this study suggests that the effects may differ. One area of research that has received little attention is the social aspect of yoga practice. While numerous randomized controlled trials have provided evidence of the physical and psychological health benefits of yoga in a variety of healthy and diseased populations [17], few have focused on or examined the influence of the social support provided in those interventions.

## 5. Conclusions

The findings of this study imply that yoga may offer diverse psychological, physical, and social effects that may make it a useful tool for healthy, sustained weight loss. The yoga practitioners reported less stress eating, reduced appetite, fewer cravings, and a shift toward healthier, more mindful eating. Yoga provided them with social support and healthy role models. The subjects believed that yoga led to physical and psychological changes that supported weight loss including increased muscle tone, improved metabolism, reduced stress, as well as increased awareness, improved mood, and greater self-acceptance and self-esteem. This weight loss experience was markedly different than past attempts, in that the weight loss was easier, and subjects felt more confident in their ability to maintain lasting weight loss.

## Figures and Tables

**Table 1:** Participant characteristics (*n* = 20).

Characteristic	Frequency (%)	
Current age, mean (SD) range	56.6 (9.4)	
	35–67	

Gender		
Female	15 (75)	

Race/ethnicity		
White/non-Hispanic	19 (95)	

Marital status		
Married/partnered	15 (75)	
Single/divorced	4 (20)	
Widowed	1 (5)	

Education		
High school/some college	2 (10)	
College degree (undergraduate)	4 (20)	
Graduate degree (M.A., J.D., M.D., or Ph.D.)	14 (70)	

Work status		
Full time	12 (60)	
Part time	3 (15)	
Retired/not employed	5 (25)	

Years of yoga practice, mean (SD) range	15.3 (13.6)	
	1–45	

Style of yoga practiced		
Iyengar	15 (75)	
Iyengar + other	2 (10)	
Other^*∗*^	3 (15)	

Weight loss in pounds, mean (SD) range^*∗∗*^	26.3 (20.2)	
	4–70	

Current BMI, mean (SD) range	27.4 (5.7)	
	17.1–41.8	

BMI category	Before yoga	After yoga
Underweight (<18.5)	1 (5)	1 (5)
Normal (18.5–24.99)	8 (40)	15 (75)
Overweight (25–29.9)	5 (25)	4 (20)
Obese (≥30)	6 (30)	1 (5)

^*∗*^Other styles included Ashtanga, Bikram, and Yin yoga.

^*∗∗*^Weight loss was reported retrospectively and is therefore approximate.

**Table 2:** Frequency of interview content coded to themes and subthemes by preyoga body mass index (BMI).

Themes and subthemes	Preyoga BMI category	
	Normal weight (*n* = 9)^*∗*^	Overweight (*n* = 11)
	Frequency (%)	
Shift toward healthy eating	7 (77.8)	11 (100)
Mindful eating	6 (66.7)	9 (81.8)
Changes in food choices	6 (66.7)	9 (81.8)
Decreased emotional or stress eating	2 (22.2)	7 (63.6)

Yoga community and culture	4 (44.4)	8 (72.7)
Role modeling healthy behaviors	2 (22.2)	4 (36.4)
Different than gym culture	4 (44.4)	6 (54.5)

Physical changes	9 (100)	11 (100)
Increased muscle tone	4 (44.4)	8 (72.7)
Change in metabolism	5 (55.6)	5 (45.5)
Effects of poses and practice on weight	9 (100)	9 (81.8)
Other physical effects contributing to weight loss	7 (77.8)	11 (100)

Psychological changes	8 (88.9)	11 (100)
Shift in mindset (health not weight)	2 (22.2)	5 (45.5)
Spirituality	5 (55.6)	4 (36.4)
Mindfulness and focus	5 (55.6)	10 (90.9)
Improved mood and emotional stability	4 (44.4)	10 (90.9)
Reduced stress	6 (66.7)	8 (72.7)
Self-esteem and self-acceptance	2 (22.2)	7 (63.6)

Different weight loss experience	4 (44.4)	10 (90.9)

*∗* includes one practitioner who was underweight, but whose themes and subthemes matched the normal weight participants.
